# Investigation on Test Mass Capture of an Inertial Sensor via Torsion Pendulum Under Limited Electrostatic Actuation for Space-Based Gravitational Wave Detection

**DOI:** 10.3390/mi17101126

**Published:** 2026-09-27

**Authors:** Yongkun Chen, Tao Yu, Huadong Li, Longqi Wang, Ke Xue, Xin Liu, Yuzhu Chen, Zhi Wang

**Affiliations:** 1 Changchun Institute of Optics, Fine Mechanics and Physics, Chinese Academy of Sciences, Changchun 130033, China; chenyongkun@ciomp.ac.cn (Y.C.); lihuadong1995@163.com (H.L.); fygoal@aliyun.com (L.W.); xuekeciomp@163.com (K.X.); liuxciomp@163.com (X.L.); chenyz_mail@163.com (Y.C.); wz070611@126.com (Z.W.); 2 University of Chinese Academy of Sciences, Beijing 100049, China

**Keywords:** test mass capture, inertial sensor, electrostatic actuation, torsion pendulum, space-based gravitational-wave detection

## Abstract

For space-based gravitational-wave detection, a released test mass in an inertial sensor may retain appreciable residual angular motion, while electrostatic actuation authority is finite. This work investigates single-axis test-mass capture about the suspension-wire axis using a torsion-pendulum platform integrating differential capacitive sensing, electrostatic actuation, and digital control in the same hardware loop. Capacitive-readout calibration, free-oscillation identification, and static torque-balance actuation calibration establish quantitative relations among test-mass motion, suspension dynamics, control voltage, and electrostatic torque, and characterize the nominal near-center actuation authority. At controller activation, the measured angular offset is approximately 15.6 mrad, and the estimated angular velocity is approximately 800 μrad/s. Under limited electrostatic actuation, the control output remains at or near saturation for approximately 110 s during the initial braking stage; the test mass then reverses direction, the output desaturates, and the response converges toward the housing center. The maximum angular displacement after activation is approximately 0.067 rad, with no further test-mass–electrode-housing contact observed. After approximately 550 s, the test mass enters a stable near-center region, where an out-of-loop autocollimator measures an angular RMS of 1.81 μrad. The corresponding command-equivalent electrostatic control torque has an RMS value of $2.6\times 10^{-11}$ N·m. These results provide a quantitative experimental characterization of single-axis test-mass capture from a large initial dynamic state under limited electrostatic actuation authority.

## 1. Introduction

In space-based gravitational-wave detectors, the test mass (TM) serves as the inertial reference for precision measurement, while the inertial sensor measures its motion relative to the surrounding spacecraft. This basic configuration is adopted in missions and mission concepts such as LISA, Taiji, and TianQin [1,2,3]. In a typical inertial sensor, the TM is enclosed by an electrode housing (EH), where opposing electrodes are used for capacitive sensing and electrostatic actuation. Differential capacitance provides the relative position and attitude of the TM, while controlled electrode voltages generate electrostatic forces and torques to regulate its motion when required [4,5,6].

During launch, the TM must be mechanically constrained and is released only after the spacecraft reaches its operating environment. Following release, the TM must be brought from its residual motion toward the nominal operating position before normal operation can be established. The released TM may retain position or attitude offsets together with nonzero translational or angular velocities. Since the available electrostatic force and torque are limited, such residual motion can drive the TM far from its nominal position and may require the actuator to operate close to saturation. This situation occurred in LISA Pathfinder (LPF), where unexpectedly large residual motion and contact with the EH were observed after the first TM release [7]. Later investigations related this behavior to the release conditions and the dynamics of the release mechanism [8]. The post-release capture process therefore involves bringing the TM toward its operating region while respecting the limited electrostatic actuation authority.

Previous studies on post-release TM regulation and capture have focused mainly on dynamic modeling and constrained control. Early work established nonlinear spacecraft dynamics for the LISA drag-free and attitude control system and investigated robust suspension-control strategies [9,10]. Subsequent studies considered the more demanding capture problem under large initial motion and limited actuation, using adaptive sliding-mode and model-predictive control approaches [11,12]. State and input constraints, together with actuator saturation, were further addressed using constrained nonlinear model predictive control and state-dependent Riccati-equation methods [13,14]. These studies have clarified several important aspects of post-release capture dynamics, particularly the influence of the initial state and actuator limits. Experimental investigations of the corresponding capture transient using actual sensing and actuation hardware, however, remain relatively limited.

Ground-based investigation of inertial-sensor dynamics and electrostatic actuation commonly relies on torsion-pendulum platforms, owing to their low torsional stiffness and high torque sensitivity. Previous studies have developed LISA-like and multi-stage torsion pendulums for inertial-sensor performance characterization and low-frequency disturbance measurements [15,16,17]. Related experiments have also addressed electrostatic actuation crosstalk, sensitivity improvement, and motion in multiple physical degrees of freedom [18,19,20]. These efforts provide the experimental basis for integrating capacitive sensing, electrostatic actuation, and closed-loop TM control in ground tests.

More recent torsion-pendulum experiments have extended toward practical sensing–actuation chains and closed-loop control. Wang et al. developed a single-TM low-frequency torsion pendulum and characterized its background torque noise and sensitivity [21]. Yang et al. investigated electrostatic actuation and angular tracking under different maximum electrostatic torques [22], while Jiang et al. demonstrated translational and rotational closed-loop operation using a two-stage torsion pendulum integrating capacitive sensing, electrostatic actuation, and digital control [23]. Wide-range electrostatic actuation electronics [24], actuation-to-sensing crosstalk [25], and translational–rotational cross-coupling [26] have also been experimentally investigated. These studies have established important capabilities in electrostatic actuation, angular regulation, integrated closed-loop control, and coupling characterization. Experimental characterization of capture transients starting from a measured large angular state under finite actuation authority, particularly those involving actuator saturation and subsequent recovery toward the operating region, remains comparatively less explored.

Against this background, this work investigates single-axis TM capture about the suspension-wire axis using a single-test-mass torsion pendulum. The experiment starts from a measured large angular offset and significant nonzero angular velocity, with the electrostatic control authority constrained by a finite voltage limit. Under these conditions, the measured transient includes actuator saturation, braking, motion reversal, desaturation, and subsequent convergence toward the EH center. The capacitive-sensing–electrostatic-actuation hardware loop provides real-time measurement of the TM angular motion and electrostatic feedback control. Capacitive-readout calibration, free-oscillation identification, and electrostatic actuation-gain calibration based on static torque balance establish quantitative relationships among sensor output, TM angular motion, suspension dynamics, control voltage, and electrostatic torque. The capture process is analyzed using the measured time histories of angle, angular velocity, and control output together with the phase-plane trajectory. After capture, the TM state is further characterized using an independent autocollimator measurement and the command-equivalent electrostatic control torque reconstructed from the controller output using the calibrated actuation gain.

The remainder of this paper is organized as follows. Section 2 introduces the torsion-pendulum setup, dynamics, capacitive sensing, electrostatic actuation, and capture-control implementation. Section 3 presents the experimental platform, system calibration, and capture results. Section 4 summarizes the work and discusses future research directions.

## 2. Experimental Principle and Control Model

The experiment focuses on TM rotation about the suspension-wire axis. The low torsional stiffness of the wire provides a sufficiently long time scale for observing the angular response of the TM under limited electrostatic actuation. At the same time, the suspension introduces a restoring torque that contributes to the measured angular dynamics and must therefore be characterized before the capture experiment. The following sections describe the single-axis dynamics, capacitive readout, electrostatic actuation, and capture controller used in the experiment.

### 2.1. Inertial-Sensor Configuration

The experimental object is a capacitive inertial-sensor assembly consisting of a test mass (TM), an electrode housing (EH), and front-end electronics (FEE). The electrode arrangement of the EH is shown in Figure 1. The hollow lightweight TM is used to reduce the rotational inertia and the load on the suspension system. Twelve electrodes are arranged around the TM to form six opposing sensing/actuation pairs.

This study uses the rotational degree of freedom about the vertical suspension-wire axis. The rotation angle θ is defined as the angular displacement of the TM about this axis relative to the EH-centered reference orientation, with the positive direction indicated in Figure 1. Four *x*-axis electrodes provide two differential-capacitance channels for measuring the TM angular motion and are also used to generate bidirectional electrostatic torque about the same axis. The TM is suspended inside the EH by a tungsten wire, while the FEE converts the differential-capacitance signals into the electrical readout used for real-time feedback. Control voltages are applied to the corresponding electrodes to produce the electrostatic torque required for capture.

### 2.2. Torsional Dynamics

The suspended TM is modeled as a single-degree-of-freedom torsional oscillator about the suspension-wire axis. As defined in Figure 1, θ denotes the TM angular displacement relative to the EH-centered reference orientation, whereas $\theta_{s}$ denotes the zero-torque orientation of the suspension wire in the same reference frame (Figure 2). The distinction between these two angles is retained because the mechanical equilibrium of the suspension does not necessarily coincide with the EH center. Neglecting cross-axis coupling and treating the suspension restoring torque as linear over the angular range considered here, the rotational dynamics are

(1) $$I\ddot{\theta }\left(t\right)+b\dot{\theta }\left(t\right)+\kappa \left[\theta \left(t\right)-\theta_{s}\right]=N_{e}\left(t\right)+N_{d}\left(t\right).$$

where I, b, and κ are the equivalent moment of inertia, damping coefficient, and torsional stiffness, respectively; $N_{e}$ is the electrostatic torque and $N_{d}$ represents equivalent disturbance torques. The term $\kappa \left(\theta -\theta_{s}\right)$ describes the restoring torque introduced by the suspension wire. Thus, changing $\theta_{s}$ changes both the mechanical equilibrium position and the restoring torque acting on the TM at a given angle. This property is used later in the static torque-balance calibration of the electrostatic actuation gain.

(2) $$\omega_{0}=\sqrt{\frac{\kappa }{I}},\;\;\zeta =\frac{b}{2\sqrt{\kappa I}}.$$

The equilibrium offset $\theta_{s}$ does not affect the natural frequency or damping ratio. The suspension parameters can therefore be identified independently from free angular oscillation, as described in Section 3.2.2.

### 2.3. Capacitive Angle Readout

The TM angle used for feedback is reconstructed from two *x*-axis differential-capacitance channels formed by the opposing electrode pairs $C_{1}/C_{3}$ and $C_{2}/C_{4}$, as shown in Figure 2. Rotation about the suspension-wire axis changes the local electrode gaps at the two measurement positions, producing opposite displacement responses in the two differential channels.

For the $C_{1}/C_{3}$ channel, let $x_{13}$ denote the local displacement and $d_{0}$ the nominal electrode gap. Under the parallel-plate approximation, the corresponding electrode differential capacitance is

(3) $$\Delta C_{13}=C_{1}-C_{3}=\frac{2\varepsilon_{0}Ax_{13}}{d_{0}^{2}-x_{13}^{2}}.$$

where $\varepsilon_{0}$ is the vacuum permittivity, A is the effective electrode area, and $d_{0}$ is the nominal electrode gap at the centered position.

Similarly, the second differential channel gives

(4) $$\Delta C_{24}=C_{2}-C_{4}=\frac{2\varepsilon_{0}Ax_{24}}{d_{0}^{2}-x_{24}^{2}}.$$

where $x_{13}$ and $x_{24}$ are the local displacements at the two measurement positions. Equations (3) and (4) describe the geometric relation between TM motion and the differential-capacitance response.

The front-end electronics convert the differential-capacitance signals into the readout voltages $V_{13}$ and $V_{24}$, which are represented as

(5) $$V_{13}=G_{13}\Delta C_{13}+V_{off,13},\;\;V_{24}=G_{24}\Delta C_{24}+V_{off,24}.$$

where $G_{13}$ and $G_{24}$ are the corresponding readout gains, and $V_{off,13}$ and $V_{off,24}$ are the voltage offsets. In the actual sensor, the voltage-to-displacement relation is influenced by the electrode geometry, alignment, parasitic capacitance, and the readout electronics. The relation used in the experiment is therefore determined directly by the end-to-end capacitive calibration described in Section 3.2.1, rather than solely from the ideal parallel-plate model. The calibrated inverse relations are then used to convert the measured voltages $V_{13}$ and $V_{24}$ into the local displacements $x_{13}$ and $x_{24}$.

Let $l_{x}$ denote the effective lever arm from the suspension-wire axis to the two measurement positions. Under the small-angle geometric approximation, the TM angle is obtained from the two calibrated local displacements as

(6) $$\hat{\theta }=\frac{x_{13}-x_{24}}{2l_{x}}.$$

The resulting capacitive angle estimate is used as the real-time feedback variable in the capture experiment. The autocollimator is independent of this feedback chain and is used as an out-of-loop optical measurement of the TM angular state.

### 2.4. Electrostatic Actuation

The four *x*-axis electrodes are used to generate the electrostatic control torque about the suspension-wire axis. Under the parallel-plate approximation, with fringe effects neglected and the TM potential taken as zero, the generalized electrostatic torque can be expressed as

(7) $$N_{e}=\frac{1}{2}\sum_{i=1}^{4}V_{i}^{2}\frac{\partial C_{i}}{\partial \theta }.$$

where $V_{i}$ and $C_{i}$ are the voltage and capacitance of the i-th electrode, respectively. For a TM rotation θ, the gaps on the two opposing sides vary approximately as $d_{0}\mp l_{x}\theta$. Differentiating the corresponding capacitances with respect to θ gives

(8) $$N_{e}=\frac{\varepsilon_{0}Al_{x}}{2}\left[\frac{V_{1}^{2}+V_{4}^{2}}{{\left(d_{0}-l_{x}\theta \right)}^{2}}-\frac{V_{2}^{2}+V_{3}^{2}}{{\left(d_{0}+l_{x}\theta \right)}^{2}}\right].$$

Equation (8) shows that the electrostatic torque is determined by both the applied electrode voltages and the instantaneous electrode gaps. The effective voltage-to-torque relation therefore varies with the TM angular position, particularly when the TM moves appreciably away from the EH center.

For bidirectional actuation, a preload voltage $V_{p}$ is combined with the signed control voltage $V_{c}$ and applied differentially to the opposing electrodes:

(9) $$\left[\begin{array}{c} V_{1}\\ V_{2}\\ V_{3}\\ V_{4} \end{array}\right]=\left[\begin{array}{c} V_{p}+V_{c}\\ V_{p}-V_{c}\\ V_{p}-V_{c}\\ V_{p}+V_{c} \end{array}\right].$$

The admissible range of $V_{c}$ is constrained by the available electrode-voltage range,

(10) $$\left|V_{c}\right|\leq V_{p}.$$

so that the commanded torque can change direction while all electrode voltages remain within the operating range of the actuation electronics.

Near the EH center, where the variation in the electrode gaps is small, the electrostatic torque can be represented locally as

(11) $$N_{e}\approx K_{v}V_{c}+\Gamma_{e}\theta .$$

where $K_{v}$ is the local control-voltage-to-torque gain and $\Gamma_{e}$ represents the angle-dependent electrostatic stiffness term. In the present work, $\Gamma_{e}$ is not independently identified, and its contribution is therefore not separately quantified in the analysis of the capture transient. The experimentally calibrated actuation parameter is $K_{v}$, which relates the signed control voltage $V_{c}$ to the control torque near the nominal operating position and is determined by static torque balance in Section 3.2.3.

In the present apparatus, the preload voltage is fixed at $V_{p}=6\;V$, and the signed control voltage is limited by

(12) $$\left|V_{c}\right|\leq 6\;V.$$

which gives an electrode-voltage range of 0–12 V. This voltage range defines the available electrostatic actuation boundary used in the capture experiment.

### 2.5. Capture Controller

The closed-loop capture uses a conventional digital PID controller. The calibrated capacitive angle $\hat{\theta }\left(k\right)$ is used as the feedback variable, and the EH center is taken as the angular reference, $\theta_{ref}=0$. At each sampling instant k, the angular error is defined as $e\left(k\right)=\theta_{ref}-\hat{\theta }\left(k\right)$, and the unsaturated signed control voltage is calculated as

(13) $$V_{c,0}\left(k\right)=K_{c}\left[e\left(k\right)+I_{e}\left(k\right)+T_{d}\frac{e\left(k\right)-e\left(k-1\right)}{T_{s}}\right].$$

where $K_{c}$ is the proportional gain, $I_{e}\left(k\right)$ is the integral contribution, $T_{d}$ is the derivative time, and $T_{s}$ is the control sampling period. The integral contribution is updated using the integral time $T_{i}$.

Because the electrostatic actuator can provide only a finite control voltage, a large initial angular state may cause the PID command to remain at the voltage limit for an extended period. Conditional-integration anti-windup is therefore used to prevent continued accumulation of the integral term during saturation. More specifically, let $\Delta I_{e}\left[k\right]$ denote the integral increment calculated at the current sampling instant. The integral state is updated when $|V_{c,0}\left[k\right]|\leq V_{c,max}$, or when the integral increment tends to return a saturated command toward the admissible range, i.e., $V_{c,0}\left[k\right]>V_{c,max}$ with $\Delta I_{e}\left[k\right]<0$, or $V_{c,0}\left[k\right]<-V_{c,max}$ with $\Delta I_{e}\left[k\right]>0$. Otherwise, the integral state is held at its previous value. The controller-output voltage command sent to the actuation stage is limited by

(14) $$V_{c}\left(k\right)=\left\{\begin{array}{ll} V_{c,max}, & V_{c,0}\left(k\right)>V_{c,max},\\ V_{c,0}\left(k\right), & \left|V_{c,0}\left(k\right)\right|\leq V_{c,max},\\ -V_{c,max}, & V_{c,0}\left(k\right)<-V_{c,max}. \end{array}\right.$$

where $V_{c,max}$ denotes the maximum magnitude of the signed control voltage. In the present experiment, $V_{c,max}=6\;V$, consistent with the actuation-voltage range defined in Section 2.4. The resulting $V_{c}$ is combined with the preload voltage $V_{p}$ according to the electrode-voltage allocation in Equation (9), so that the controller operates within the 0–12 V range of each actuation electrode.

Figure 3 shows the resulting control implementation, from the calibrated capacitive angle and PID calculation to anti-windup, output limiting, and electrode-voltage allocation.

## 3. Experiments and Results

The experiments were carried out on the single-TM torsion-pendulum platform shown in Figure 4. System calibration and identification are presented first, followed by the closed-loop capture test.

### 3.1. Experimental Platform

The vacuum chamber reduces air damping and airflow disturbances, while an internal permalloy shield reduces magnetic-field effects. The pressure is approximately 7 × 10^−2^ Pa and the laboratory temperature variation is maintained within ±0.1 °C.

The suspension assembly consists of upper and lower tungsten fibers, a magnetic damper, a measurement prism, a leveling/limiting mount, and the TM. A four-degree-of-freedom adjustment mechanism at the upper end sets the TM position relative to the EH, and the magnetic damper suppresses pendular modes.

An external autocollimator measures TM rotation through a suspension-mounted prism and provides an independent post-capture angular measurement.

The principal apparatus parameters are summarized in Table 1; all listed values are nominal design values for the present apparatus.

### 3.2. Calibration and Identification

Before the capture experiment, the sensing, suspension dynamics, and electrostatic actuation are characterized separately. Capacitive calibration establishes the relation between the electrical readout and TM displacement. Free-oscillation measurements are then used to identify the torsional dynamics of the suspension, and the identified restoring torque provides the mechanical reference for electrostatic actuation calibration. Finally, static torque balance is used to determine the control-voltage-to-torque gain near the EH center. These measurements provide the parameters and conversion relations used in the subsequent capture experiment.

#### 3.2.1. Capacitive Calibration

Before the TM is suspended, it is fixed and the EH is translated stepwise relative to it using a six-degree-of-freedom positioning stage. Twenty-nine nominal positions are used for each channel, covering approximately ±2.1 mm for the *x*-axis channels and ±1.4 mm for the *y*- and *z*-axis channels.

At each position, a 12 s steady-state record sampled at 10 Hz is averaged to obtain one calibration point, and the resulting points are fitted with the third-order voltage–displacement polynomial in Equation (15).

(15) $$V_{i}\left(x_{i}\right)=a_{3,i}x_{i}^{3}+a_{2,i}x_{i}^{2}+a_{1,i}x_{i}+a_{0,i}.$$

where $V_{i}$ and $x_{i}$ denote the readout voltage and displacement of the i-th sensing channel, respectively. The fitted coefficients are listed in Table 2, and the calibration results are shown in Figure 5. The $x_{1}$ and $x_{2}$ channels correspond to the $C_{1}/C_{3}$ and $C_{2}/C_{4}$ differential-capacitance channels used for angular sensing, while the remaining channels are used to assist TM centering.

The third-order form is selected because the ideal differential-capacitance response contains a cubic term as the leading nonlinear correction to the linear response. A general third-order polynomial is therefore used as an end-to-end empirical calibration model for the complete sensing chain.

The coefficients are estimated by ordinary least-squares regression, with 95% confidence intervals obtained from the regression covariance matrix. Fit quality is reported using the RMSE and adjusted $R^{2}$.

The fitted relations are inverted during the experiment to convert the measured capacitive voltages into local TM displacements. The two *x*-axis displacement estimates are then combined according to Equation (6) to obtain the angular feedback used for capture.

#### 3.2.2. Suspension Identification

With electrostatic actuation disabled, the angular motion about the suspension-wire axis is described as a weakly damped free oscillation:

(16) $$\theta \left(t\right)=A_{0}e^{-\zeta \omega_{0}t}cos\left(\omega_{d}t+\phi_{0}\right)+\theta_{b}.$$

where $A_{0}$, ζ, $\omega_{0}$, $\omega_{d}$, $\varphi_{0}$, and $\theta_{b}$ denote the initial oscillation amplitude, damping ratio, undamped angular frequency, damped angular frequency, initial phase, and equilibrium offset, respectively. For the weak damping of the present suspension, $\omega_{d}\approx \omega_{0}$.

The suspension parameters are identified from a free angular-decay record acquired at 10 Hz with electrostatic actuation disabled, as shown in Figure 6. The damped frequency determined from the measured periods is $f_{d}=1.885\;\mathrm{mHz}$. A logarithmic regression of the oscillation envelope gives an effective damping ratio of $\zeta =(1.13\pm 0.01)\times 10^{-3}$, where the stated uncertainty is the half-width of the 95% regression confidence interval; the fit gives $R^{2}\approx 0.998$.

Using the nominal moment of inertia $I=44\times 10^{-6}\;\mathrm{kg}\;m^{2}$ gives $\kappa =(6.170\pm 0.005)\times 10^{-9}\;N\;m/\mathrm{rad}$. The identified parameters provide the mechanical reference for the electrostatic-actuation calibration in Section 3.2.3.

#### 3.2.3. Actuation Gain Calibration

The local control-voltage-to-torque gain $K_{v}$ is calibrated near the EH center using static torque balance. During the calibration, the TM is maintained close to the center while the upper suspension point is rotated stepwise to change the zero-torque orientation $\theta_{s}$ of the suspension wire. After each adjustment, the system is allowed to reach equilibrium and the steady control voltage is averaged. Changing $\theta_{s}$ changes the suspension restoring torque while keeping the TM close to the nominal sensing and actuation position. The calibration comprises 41 step settings covering −2° to +2° in 0.2° increments, including forward and return points; at each setting, a 200 s steady-state interval (2000 samples at 10 Hz) is averaged.

At static equilibrium, the electrostatic torque balances the restoring torque of the suspension. With the TM maintained close to the EH center so that the residual angle is neglected, the torque balance gives

(17) $$K_{v}V_{c}\approx \kappa \theta_{s}.$$

where the sign convention is consistent with the torsional dynamics defined in Section 2.2.

Figure 7 shows the measured steady control voltage as a function of the suspension zero-torque orientation $\theta_{s}$. The data exhibit an approximately linear dependence with a small intercept. A linear regression including an intercept gives a coefficient of determination of 0.99981. The fitted slope is

(18) $$\theta_{s}=K_{\theta V}V_{c},\;\;K_{\theta V}=(0.02866\pm 0.00013)\;rad/V.$$

Combining this slope with the torsional stiffness κ identified in Section 3.2.2 gives the near-center control-voltage-to-torque gain

(19) $$K_{v}=\kappa K_{\theta V}=(1.768\pm 0.008)\times 10^{-10}\;N\cdot m/V.$$

and hence the local control-voltage-to-torque relation used to characterize the available actuation

(20) $$N_{eq}\left(t\right)=K_{v}V_{c}\left(t\right).$$

With $K_{v}=(1.768\pm 0.008)\times 10^{-10}\;N\;m/V$ and $|V_{c}|\leq 6\;V$, the corresponding nominal maximum control torque near the EH center is approximately $1.06\times 10^{-9}\;N\;m$. For $I=44\times 10^{-6}\;kg\;m^{2}$, this corresponds to a nominal angular acceleration of $2.4\times 10^{-5}\;rad/s^{2}$ ($24.1\;\mu rad/s^{2}$). These values characterize the local electrostatic actuation scale available near the nominal operating position. Accordingly, the resulting gain should be interpreted as a near-center actuation reference rather than a full-angle torque characterization of the large-angle capture trajectory. A refined quantitative description over a wider angular range would require angle-dependent electrostatic-actuation characterization together with the identified suspension dynamics.

### 3.3. Capture Test

#### 3.3.1. Initial Condition and Setup

The capture experiment uses the calibrated *x*-axis differential-capacitance channels for real-time angular feedback. The capacitive-sensing acquisition and PID computation are executed within the same 10 Hz software loop, such that each newly acquired capacitive-angle sample is used for one controller update. The principal sensing, control, and actuation implementation parameters used in the capture experiment are summarized in Table 3.

Before the capture test, the suspension zero-torque orientation was set to approximately $\theta_{s}=+2{}^{\circ}$(0.0349 rad) relative to the EH-centered reference orientation, with the positive direction defined as in Figure 1. According to Equation (1), the suspension torque is $N_{s}=-\kappa \left(\theta -\theta_{s}\right)$. Therefore, at the nominal EH-centered position (θ=0), the suspension torque is positive and tends to drive the TM toward the mechanical zero-torque orientation rather than acting as a braking torque. At controller activation, $\theta_{0}=15.6$ mrad is still smaller than $\theta_{s}$; thus, the suspension torque initially acts in the same direction as the outward TM motion.

To generate the initial condition for capture, a large angular motion was first produced by open-loop electrostatic actuation. During this initial-state preparation, the TM contacted the EH before controller activation. Following the contact, as the TM moved back toward the center, additional open-loop electrostatic actuation was applied to shape the subsequent trajectory. The feedback controller was then activated when the measured angular state reached the selected initial condition. Thus, the TM–EH contact belongs to the preceding initial-state preparation stage and is outside the controlled capture interval analyzed below.

At controller activation, the measured angular offset is approximately 15.6 mrad, with an estimated angular velocity of approximately 800 μrad/s. These two quantities are obtained from the same measured trajectory rather than prescribed independently. The angular velocity used for transient monitoring and phase-plane analysis is obtained from the measured angle by numerical differentiation followed by smoothing. This initial state is of the same order as the large post-release angular motion reported for LPF [7,8].

#### 3.3.2. Capture Transient

Figure 8a shows the phase-plane trajectory including the preceding open-loop initial-state preparation and the subsequent controlled capture. The TM–EH contact marked in the phase plane occurs before controller activation and is therefore not part of the controlled capture interval. Figure 8b shows the estimated rotation angle, estimated angular velocity, and control voltage during the first 700 s after controller activation, with t=0 defined at controller activation. Immediately after controller activation, the saturated electrostatic control torque acts opposite to the initial outward motion. Because the initial angle is smaller than the suspension zero-torque orientation ($\theta_{0}<\theta_{s}$), the suspension torque is initially positive and therefore opposes, rather than assists, the electrostatic braking.

At approximately 110 s, the angular velocity first reaches zero and the TM reaches the turning point of the trajectory. The maximum angular displacement is approximately 0.067 rad, corresponding to an additional excursion of about 51.4 mrad after controller activation. The angular velocity then changes sign and the TM begins to move back toward the EH center.

As the TM returns toward the center, the control voltage leaves saturation and closed-loop regulation continues within the available actuation range. No further TM–EH contact is observed after controller activation, and the TM reaches a stable near-center region at approximately 550 s.

Quantitatively, the approximately 110 s interval at or near the saturation limit accounts for about 20% of the approximately 550 s elapsed before the TM enters the stable near-center region. The maximum angular displacement of approximately 0.067 rad is about 4.3 times the 15.6 mrad angular offset at controller activation, indicating a substantial additional outward excursion before motion reversal during the finite-actuation capture process. These results characterize the transition from an actuation-limited large-motion transient to stable near-center regulation in the present ground-based apparatus.

The present experiment demonstrates successful capture for one measured large initial state under the tested actuation limit. Accordingly, the present data do not establish a full capture-feasibility boundary or statistical repeatability.

#### 3.3.3. Post Capture Steady State Characterization

After the TM reaches the stable near-center region, its angular motion is independently measured using the external autocollimator, while the same feedback, actuation, and controller settings are maintained. Figure 9a shows the post-capture angular fluctuation measured by the autocollimator. The angular fluctuation remains mainly within ±6 μrad, with an RMS value of 1.81 μrad.

The steady-state controller output is converted to a command-equivalent electrostatic control torque using the near-center static actuation gain $K_{v}$ calibrated in Section 3.2.3. Figure 9b shows the corresponding command-equivalent control-torque fluctuation in the time domain. The command-equivalent torque remains mainly within $\pm 1\times 10^{-10}\;N\;m$, with an RMS value of $2.6\times 10^{-11}\;N\;m$.

Taken together, the post-capture measurements characterize the near-center TM motion and the corresponding actuator effort. The command-equivalent control torque reflects the actuator effort requested by the controller to maintain the captured state; its RMS value corresponds to about 2.5% of the nominal near-center maximum control torque of $1.06\times 10^{-9}$ N·m. This reduced control demand is consistent with the transition from the initial actuation-limited capture transient to near-center closed-loop regulation.

## 4. Conclusions and Outlook

This work investigates single-axis TM capture using a single-test-mass torsion pendulum under limited electrostatic actuation. Capacitive-readout calibration, free-oscillation identification, and static torque-balance calibration establish the quantitative relations among TM angular motion, suspension dynamics, control voltage, and electrostatic torque. Based on these calibrated relations, a closed-loop capture experiment is performed from a measured large angular offset and significant nonzero angular velocity.

At controller activation, the angular offset and angular velocity are approximately 15.6 mrad and 800 μrad/s, respectively. The control output remains at or near saturation for about 110 s during the initial braking stage, followed by motion reversal and desaturation as the TM returns toward the EH center. The maximum angle after activation is approximately 0.067 rad, with no further TM–EH contact observed. A stable near-center state is reached after about 550 s. In the post-capture state, the out-of-loop autocollimator measures an angular RMS of 1.81 μrad, while the command-equivalent electrostatic control torque remains mainly within $\pm 1\times 10^{-10}\;N\;m$, with an RMS value of $2.6\times 10^{-11}\;N\;m$. These results characterize both the capture transient under finite electrostatic actuation and the subsequent near-center state.

The present results are obtained under the current ground-based single-axis torsion-pendulum configuration, in which suspension restoring stiffness contributes to the TM dynamics, and cannot be directly extrapolated to the multi-degree-of-freedom dynamics or in-orbit actuation conditions of a freely floating TM. Future work will extend the electrostatic-actuation characterization over a wider angular range and combine it with the identified suspension dynamics for a refined quantitative interpretation of large-angle capture transients. Systematic capture tests covering different initial angular offsets, angular velocities, and actuation limits will also be conducted to characterize the capture-feasibility region, while repeated tests under comparable initial conditions will be used to quantify capture repeatability. The platform will also be extended toward multi-degree-of-freedom experiments incorporating translational–rotational coupling, electrostatic stiffness, and multi-channel actuation constraints.

## Figures and Tables

**Figure 1:**
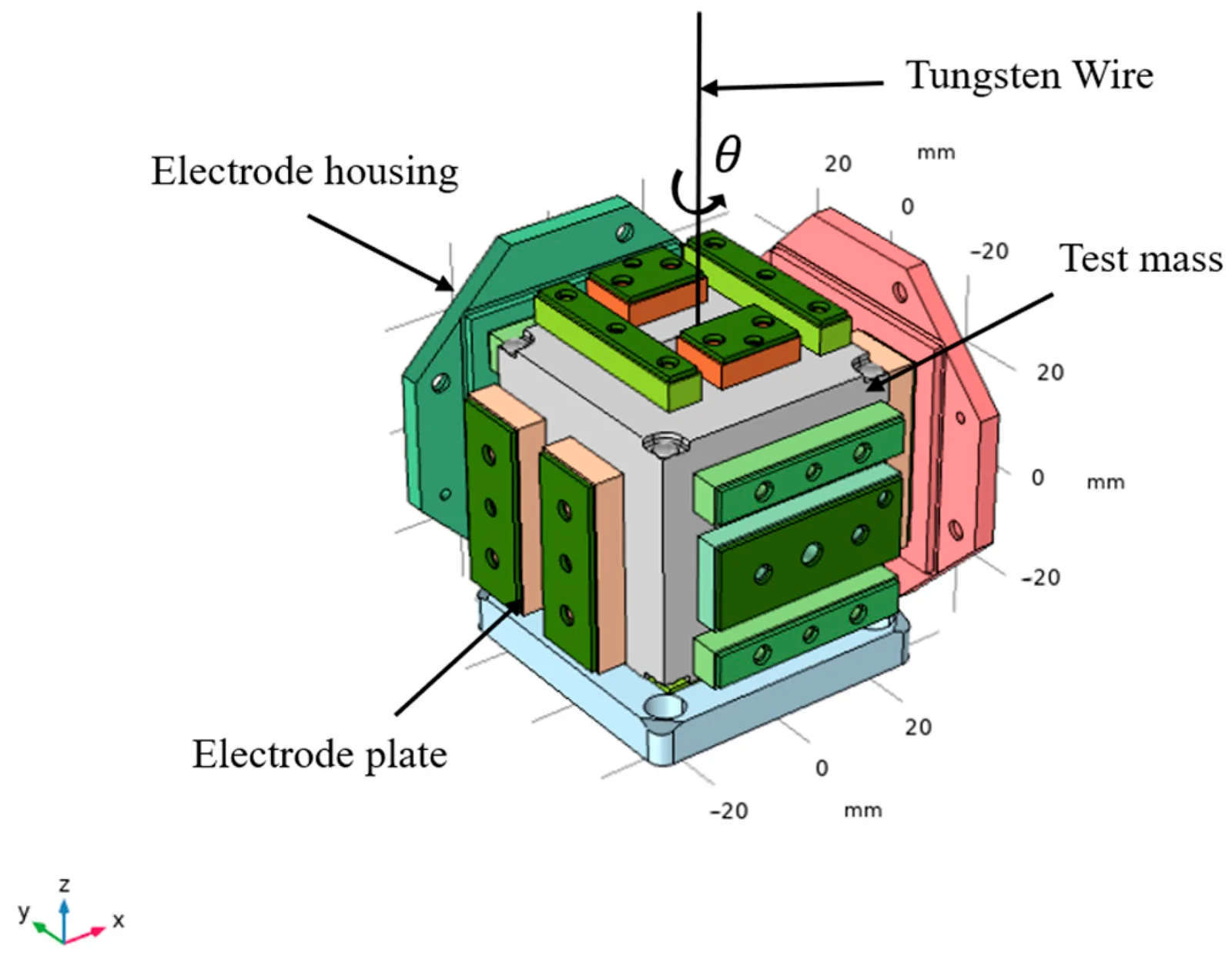
Structure of the inertial-sensor electrode housing. The TM is suspended by the vertical tungsten wire, which defines the rotation axis investigated in this work. The angle θ denotes the TM angular displacement about this axis relative to the EH-centered reference orientation, and the arrow indicates the positive direction.

**Figure 2:**
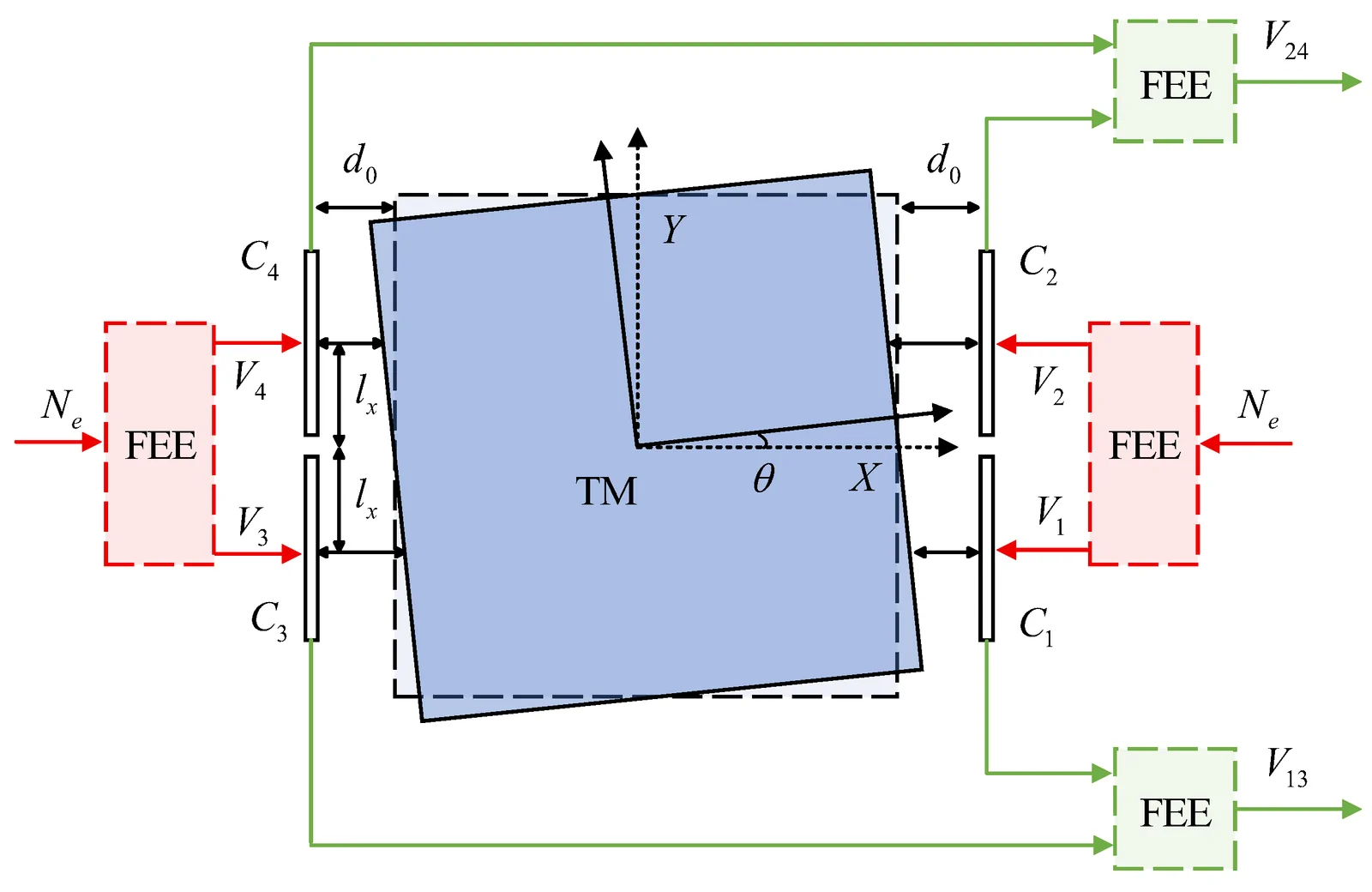
Schematic of capacitive sensing and electrostatic actuation. FEE denotes front-end electronics.

**Figure 3:**
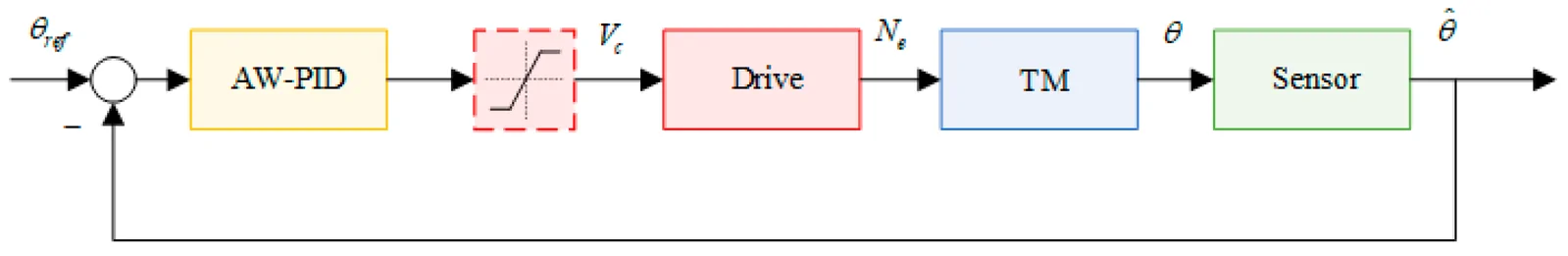
Block diagram of the capture-control implementation.

**Figure 4:**
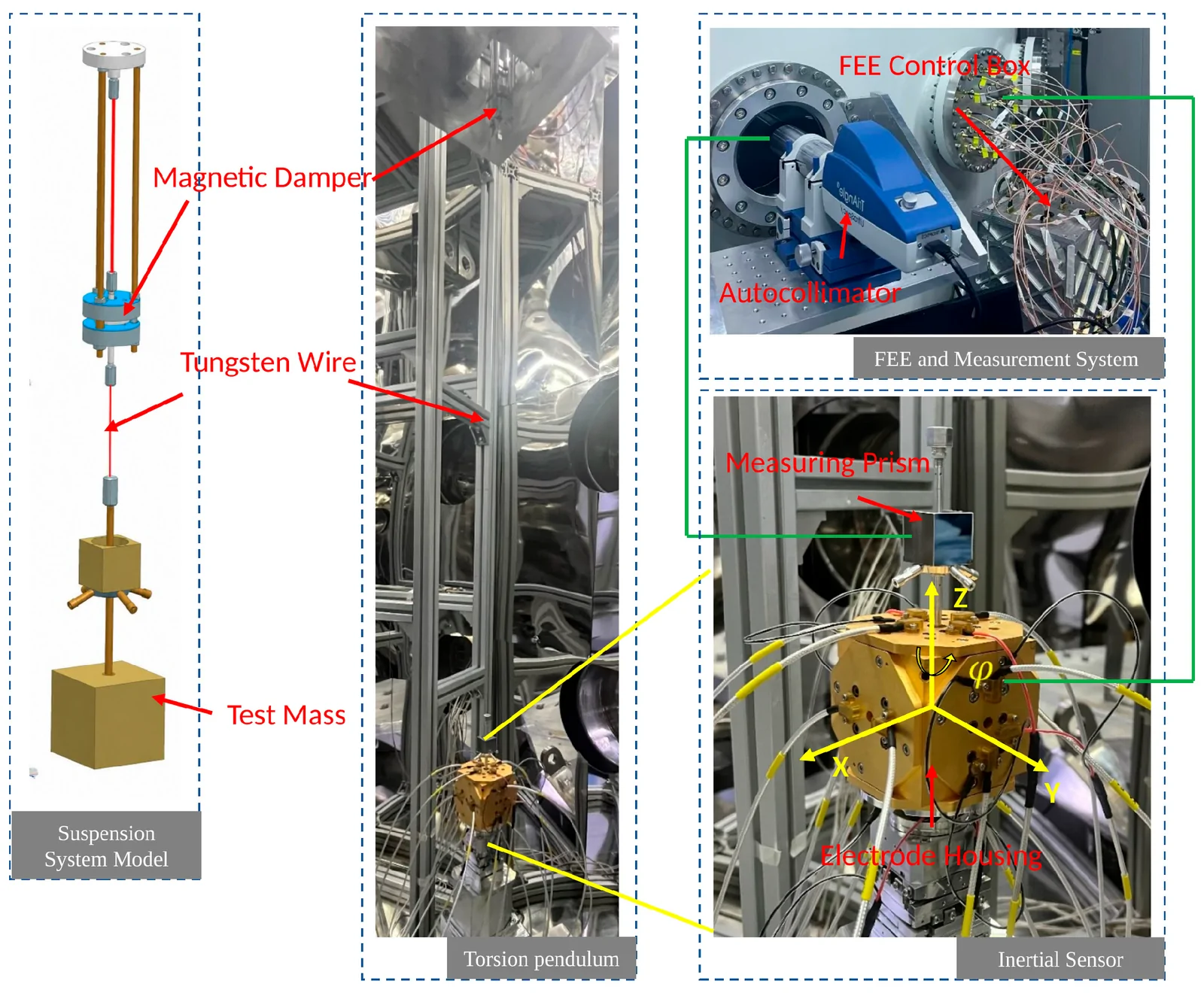
Experimental platform and measurement–control system.

**Figure 5:**
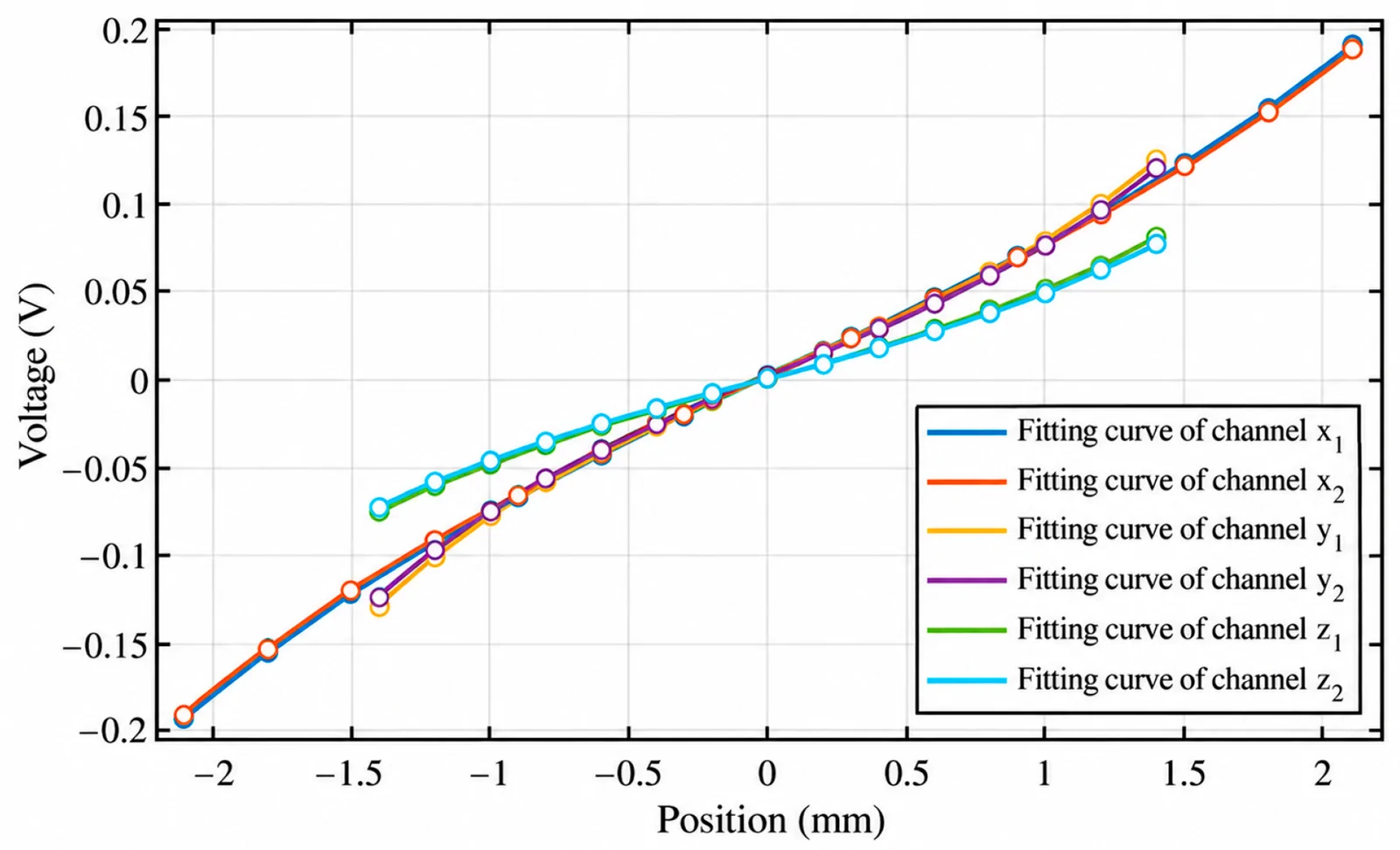
Calibration data and polynomial fits for the six capacitive-sensing channels.

**Figure 6:**
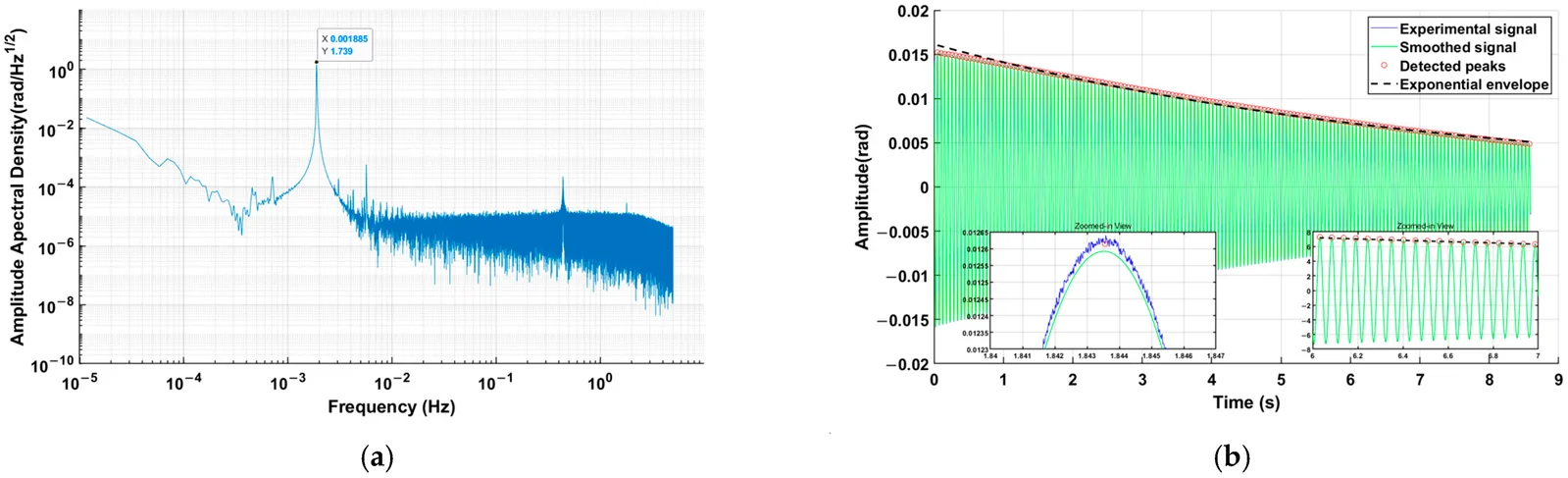
(**a**) Amplitude spectral density of the free oscillation. (**b**) Time-domain envelope fit of the free oscillation.

**Figure 7:**
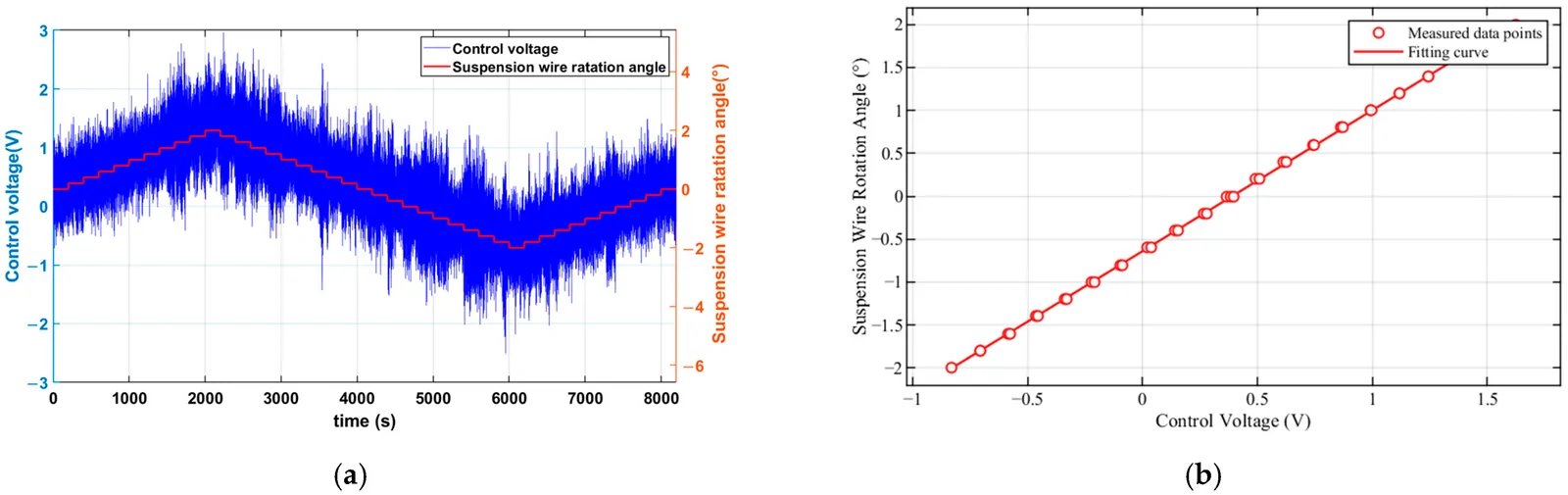
(**a**) Actuation-calibration data. (**b**) Linear fit of the actuation-calibration data.

**Figure 8:**
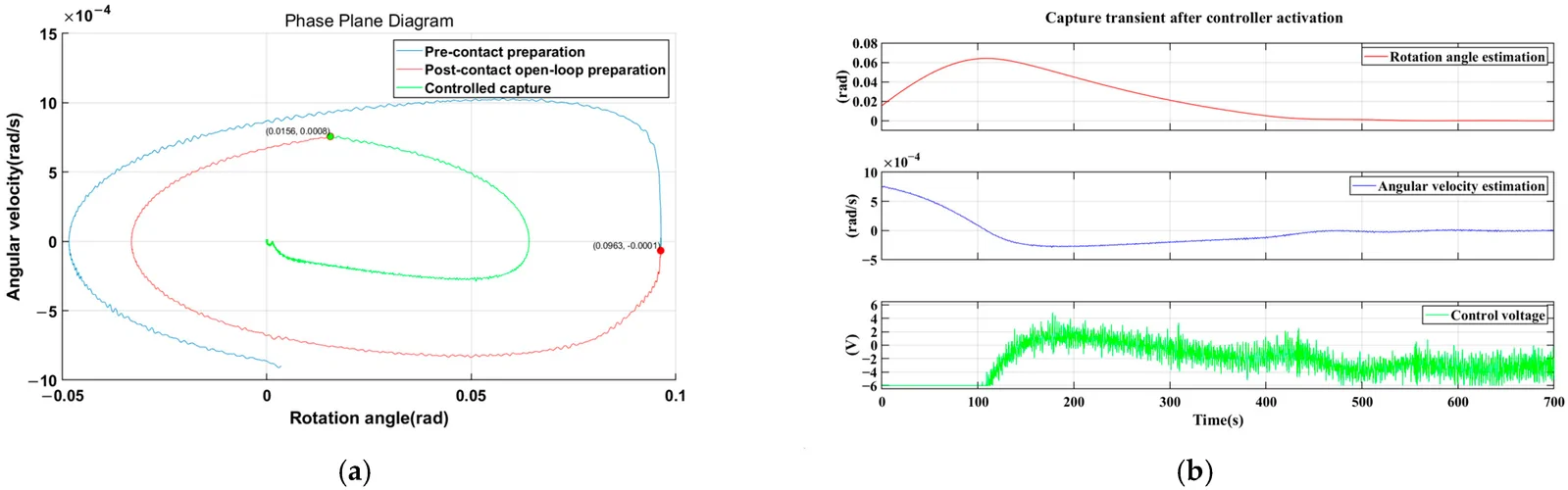
(**a**) Phase-plane trajectory showing the preceding open-loop initial-state preparation and the subsequent controlled capture. The TM–EH contact occurs before controller activation, and the controlled capture begins at the point labeled “Controller ON”. (**b**) Time histories of the estimated rotation angle, estimated angular velocity, and control voltage during the first 700 s after controller activation, with *t* = 0 defined at controller activation.

**Figure 9:**
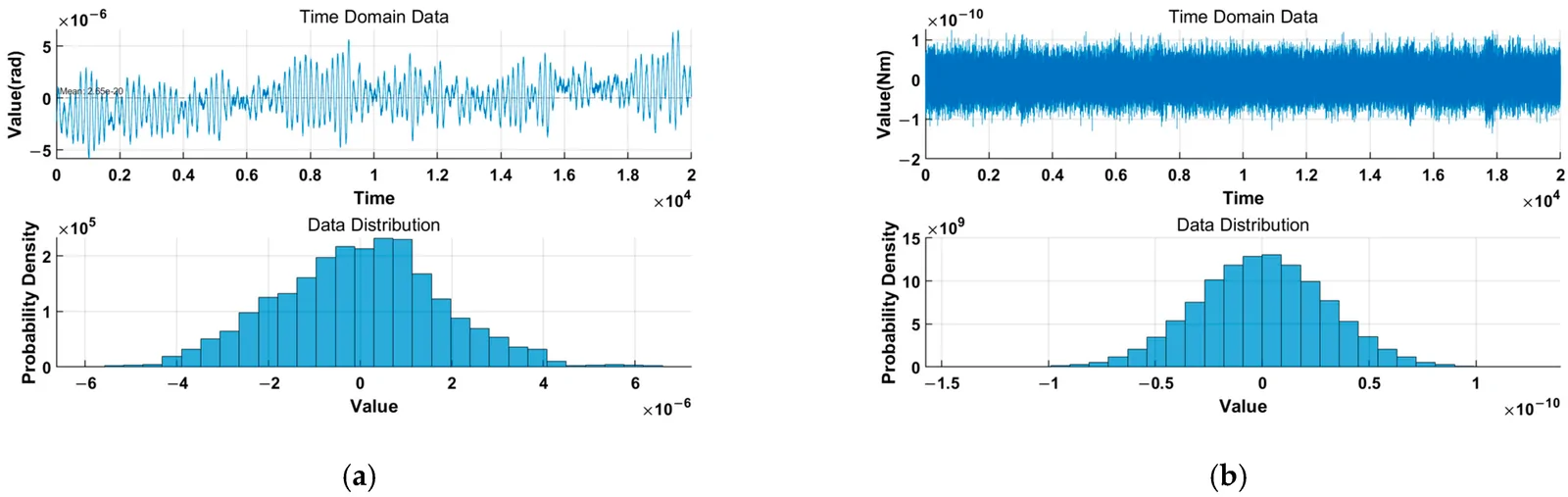
(**a**) Post-capture angular fluctuation measured by the autocollimator. (**b**) Post-capture command-equivalent electrostatic control torque fluctuation in the time domain.

**Table 1:** Main parameters of the single-test-mass torsion-pendulum apparatus.

Parameter	Symbol	Value	Unit
Upper tungsten-fiber length	*l*_1_	0.14	m
Upper tungsten-fiber diameter	*d*_1_	150	μm
Lower tungsten-fiber length	*l*_2_	1.00	m
Lower tungsten-fiber diameter	*d*_2_	25	μm
TM	*m*	107	g
TM size	*a*	64	mm
*x*-axis electrode area	*A*	522	mm^2^
*x*-axis equivalent lever arm	*l_x_*	10.7	mm
Moment of inertia about the suspension axis	*I*	44 × 10^−6^	kg·m^2^
Nominal electrode gap	*d*_0_	4	mm

**Table 2:** Third-order polynomial calibration results for the capacitive-sensing channels.

Channel	*a*_3_ (V/mm^3^)	*a*_2_ (V/mm^2^)	*a*_1_ (V/mm)	*a*_0_ (V)	RMSE (V)	Adjusted *R*^2^
*x*_1_	0.00434 ± 0.00012	−0.00082 ± 0.00013	0.07286 ± 0.00036	0.00117 ± 0.00026	4.61 × 10^−4^	0.999980
*x*_2_	0.00428 ± 0.00012	−0.00084 ± 0.00013	0.07188 ± 0.00035	0.00121 ± 0.00025	4.55 × 10^−4^	0.999980
*y*_1_	0.01195 ± 0.00033	−0.00194 ± 0.00024	0.06732 ± 0.00044	0.00123 ± 0.00021	3.80 × 10^−4^	0.999967
*y*_2_	0.01155 ± 0.00032	−0.00194 ± 0.00023	0.06497 ± 0.00043	0.00124 ± 0.00021	3.68 × 10^−4^	0.999967
*z*_1_	0.00586 ± 0.00013	0.00112 ± 0.00009	0.04457 ± 0.00017	−0.00072 ± 0.00008	1.43 × 10^−4^	0.999989
*z*_2_	0.00554 ± 0.00011	0.00052 ± 0.00008	0.04284 ± 0.00014	−0.00033 ± 0.00007	1.24 × 10^−4^	0.999991

**Table 3:** Principal sensing and digital-control implementation parameters.

Parameter	Value/Implementation
Control sampling period	0.1 s
Capacitive-sensing update rate	10 Hz
Controller execution frequency	10 Hz
ADC	AD7712, 24 bit
DAC	AD5791, 20 bit
Actuation-electronics bandwidth	5 Hz
Derivative filtering	None
PID controller gain *K*_c_	40
Integral time *T*_i_	3600 s
Derivative time *T*_d_	720 s
Signed control-voltage limit *V*_c,max_	6 V
Preload voltage *V*_p_	6 V
Electrode-voltage range	0–12 V

## Data Availability

The data supporting the findings of this study are available from the corresponding author upon reasonable request.
